# Role of Novel Multidrug Efflux Pump Involved in Drug Resistance in *Klebsiella pneumoniae*

**DOI:** 10.1371/journal.pone.0096288

**Published:** 2014-05-13

**Authors:** Vijaya Bharathi Srinivasan, Bharat Bhushan Singh, Nitesh Priyadarshi, Neeraj Kumar Chauhan, Govindan Rajamohan

**Affiliations:** Bacterial signaling and Drug Resistance Laboratory, Council of Scientific Industrial Research- Institute of Microbial Technology, Chandigarh, India; Université d’Auvergne Clermont 1, France

## Abstract

**Background:**

Multidrug resistant *Klebsiella pneumoniae* have caused major therapeutic problems worldwide due to the emergence of the extended-spectrum β-lactamase producing strains. Although there are >10 major facilitator super family (MFS) efflux pumps annotated in the genome sequence of the *K. pneumoniae* bacillus, apparently less is known about their physiological relevance.

**Principal Findings:**

Insertional inactivation of *kpnGH* resulting in increased susceptibility to antibiotics such as azithromycin, ceftazidime, ciprofloxacin, ertapenem, erythromycin, gentamicin, imipenem, ticarcillin, norfloxacin, polymyxin-B, piperacillin, spectinomycin, tobramycin and streptomycin, including dyes and detergents such as ethidium bromide, acriflavine, deoxycholate, sodium dodecyl sulphate, and disinfectants benzalkonium chloride, chlorhexidine and triclosan signifies the wide substrate specificity of the transporter in *K. pneumoniae*. Growth inactivation and direct fluorimetric efflux assays provide evidence that *kpnGH* mediates antimicrobial resistance by active extrusion in *K. pneumoniae*. The *kpnGH* isogenic mutant displayed decreased tolerance to cell envelope stressors emphasizing its added role in *K. pneumoniae* physiology.

**Conclusions and Significance:**

The MFS efflux pump KpnGH involves in crucial physiological functions besides being an intrinsic resistance determinant in *K. pneumoniae*.

## Introduction

Drug efflux, in particular multidrug efflux, is a serious problem to drug therapy as they efflux broad range of substrates including clinically relevant antimicrobial agents [1], [2], [3], [4], [5], [6]. Nosocomial pathogen *Klebsiella pneumoniae* is a Gram-negative bacillus associated with healthcare infections, known to cause variety of diseases in humans with significant morbidity and mortality [7]. *K. pneumoniae* causes various serious illnesses and the problem is aggravated due to its propensity to acquire multidrug resistance determinants [8], [9]. Recently hypervirulent, hyperviscous *K. pneumoniae* NTUH-K2044, that belongs to the K1 serotype, known to cause pyogenic liver abscess, sometimes complicated by endophthalmitis or meningitis, has emerged in Taiwan, Singapore, Korea and other Asian countries [10]. Analysis of the available genome sequence of different *K. pneumoniae* isolates from NCBI database www.ncbi.nlm.nih.gov revealed that more than 10% of total genes were annotated as transporter proteins or efflux pumps, of which, to date only few have been studied and demonstrated to have a role in drug resistance. The 5.2-Mb genome of *K. pneumoniae* strain, NTUH-K2044 (encoding 4,992 proteins, GC content: 57.7%) is reported to harbor >15 open reading frames encoding for putative efflux pumps from different families (accession number NC_012731) [10]. The efflux systems functionally characterized in *K. pneumoniae* so far include *acrAB* and *kexD* from resistance/nodulation/cell division (RND), family [11], [12], [13]; the *kdeA* efflux gene from multi drug and toxic compound extrusion (MATE) family [14]; the *kmrA* gene from major facilitator super family (MFS) family [15]; and *kpnEF* from small multidrug resistance (SMR) family [16]. In Gram-negative bacteria, a subset of inner membrane proteins in the MFS act as efflux pumps to decrease the intracellular concentrations of multiple toxic substrates and confer multidrug resistance [17], [18]. The MFS type of transporters perhaps the most largest and diverse among all the efflux super families are found in all kingdoms of life. Well-studied examples such as QacA and NorA of *Staphylococcus aureus* and SmvA of *Salmonella enterica serovar* Typhimurium belong to the latter family [19], [20], [21]. Analysis of the *K. pneumoniae* NTUH-K2044 genome reveals the presence of a novel two component efflux pump operon, an *emrAB* homolog that belongs to MFS super family; whose functions have remained completely unexplored so far [10]. The objective of the present study was to investigate the role of putative MFS efflux system, an *emrAB* homolog (denoted *kpnGH*) in *K. pneumoniae* with respect to cellular physiology and broad spectrum antimicrobial resistance.

## Materials and Methods

### Bacterial Strains, Plasmids and Media

*K. pneumoniae* NTUH-K2044 (a strain that resulted in pyogenic liver abscess in a 66 year old patient) was kindly provided by Dr. Jin Town Wang of the National Taiwan University Hospital, Taipei, Taiwan [10]. *E. coli SM10* lambda pir and pUT-Km was used to create isogenic mutants. Genomic DNA, plasmid, restriction digestion, DNA elution (Qiagen), ligation, transformation, conjugation, DNA sequencing (Applied Biosystems) were performed as previously described [22], [23], [24]. Primers used in the present study were custom-synthesized (Eurofins MWG operons, Germany).

### Cloning of *kpnGH* in Hyper Susceptible *E. coli* Strain KAM32

The putative efflux genes *kpnG* and *kpnH* were amplified by standard PCR protocol using primers *kpnG*-F; *kpnG*-R and *kpnH*-F; *kpnH*-R (Table 1) respectively and cloned into *EcoR*I and *Pst*I (New England Biolabs, MA, USA) site of pUC18. The resulting recombinant plasmid p*kpnG*, p*kpnH* and p*kpnGH* were transformed into *E. coli* KAM32 (Δ*acrAB* and Δ*ydhE*) for functional characterization.

**Table 1:** Primers used in this study.

Primer name	Primer sequences (5′–3′)
Δ*kpnGH*-F	GATATCTATGGCGATGACGTGAAATACACCGGTAA
Δ*kpnGH*-R	GATCAGCGGTCCGGCGACGATCCCCTGAAT
*kpnG*-F	CATAGGATCCATGAGTGCAAATGCGGAGAGCCAAACC
*kpnG*-R	ATTACTCGAGTTATCCGGCGTTGGCCTGAATGATCTC
*kpnH*-F	AGATCATATGCAACAGCAAAAACCCCTGGAAGGCGCGCAGCTGGTC
*kpnH*-R	ATTAGGATCCAGACCCGGAGGTCCCTTTATGTGAGGCTTAGTG
primer R-1	TATAGGATCCATTAACGAGACATTATTTATGGCGCTGATT
primer R-2	ATTAGAATTCGGTACTTTTCAGCAGGATGGCTCAGCATAGT

### Construction of the *kpnGH* Mutant in *K. pneumoniae*

The putative *emrAB* homolog, KP1_4279/KP1_4280 (designated *kpnGH*) is located starting from nucleotides 4108112 to 4109284 bps (*kpnG*: 1173 bp, 390 aa, 42.59 Kda and *kpnH*: 1536 bp, 511 aa, 55.589 Kda) in *K. pneumoniae* [10]. A 720 bp internal fragment was amplified by PCR using Δ*kpnGH*-F and Δ*kpnGH*-R primers and cloned in *EcoR*I digested pUT-Km and transformed into *E. coli SM10* lambda pir strain *(thi thr leu tonA lacY supE recA::RP4-2-Tc::Mu Km λpir)*. The recombinant plasmid pUT-*kpnGH* was mobilized into recipient *K. pneumoniae* from donor *E. coli SM10* lambda pir as described previously to create inactivation (insertional inactivation means the operon is present on the chromosome but is disrupted, therefore the *kpnGH* efflux pump is non-functional) in *kpnGH* [22], [23]. The intact *kpnGH* operon along with its promoter was amplified with primer R-1 and primer R-2 and cloned into pCRIITOPO-CAT. The resulting construct was electroporated into Δ*kpnGH* and selected on LB agar plates supplemented with 50 µg/mL kanamycin and 100 µg/mL chloramphenicol to get the transcomplemented strain Δ*kpnGH*Ω*kpnGH*.

### Bacterial Growth Curves and Growth Inactivation Assay

The growth kinetics of WT (control strain: *K. pneumoniae*), Δ*kpnGH* and Δ *kpnGH*Ω*kpnGH* were monitored in LB at different pH (5.0, 6.0, 7.0, 8.0, 10.0 and 12.0). Optical densities were measured for 18 h at 37°C shaking using Bioscreen C automated growth analysis system (Labsystems, Helsinki, Finland) at 600 nm and automatically recorded for each well after every 30 mins. The WT, Δ*kpnGH* and Δ*kpnGH*Ω*kpnGH* cultures at mid log phase (OD_600 nm_ = 0.01) were inoculated into LB broth containing antibiotic such as cefepime (0.1 µg/ml), tetracycline (0.01 µg/ml), ethidium bromide, EtBr (2 µg/ml), rhodamine (2 µg/ml), safranine (2 µg/ml) in independent experiments either alone or with efflux pump inhibitors, protonophores carbonyl cyanide 3-chlorophenylhydrazone; CCCP 2.5 µg/ml; 2, 4-Dintrophenol (2, 4 DNP); 2.5 µg/ml calcium channel blocker verampamil; 2.5 µg/ml and plant alkaloid reserpine; 2.5 µg/ml, (Sigma, St. Louis, MO) [23]. The growth at 37°C was analysed by measuring the absorbance at OD_600 nm_ periodically for 18 h in Bioscreen C automated growth analysis system (Labsystems, Helsinki, Finland). The experiment was performed with freshly autoclaved medium in triplicates at least three independent times.

### Motility Assay

The motility assays were done as reported before [23], where LB grown *K. pneumoniae* cultures (OD_600nm_ = 1.0) were inoculated with a toothpick on LB plates with 0.25%, 0.45% and 0.7% agar and incubated for 10 hrs at 37°C. In this growth medium bacteria can swim through the soft agar and produce a halo. The diameter of the halo is a measure of the ability to swim.

### Crystal Violet Binding Assay

The crystal violet binding assay was done as described before [23].

### Various Stress Challenge Assays

The WT, Δ*kpnGH*, Δ*kpnGH*Ω*kpnGH* were plated onto LB agar plates containing different substrates at varied concentrations such as bile (0.2, 0.5, 0.75, 1.0, 2.0%), sodium chloride (NaCl) (0.075, 0.15, 0.25, 0.5, 0.75, 1 and 2M), acriflavine (0.5, 4, 16, 64, 256, 512 µg/ml), acridine orange (0.5, 4, 16, 64, 256, 512 µg/ml), safranine (0.5, 4, 16, 64, 256, 512 µg/ml), rhodamine (0.5, 4, 16, 64, 256, 512 µg/ml), EtBr (0.5, 4, 16, 64, 256, 512 µg/ml), sodium dodecyl sulphate, SDS (1024, 2048, 4096, 8192, 16834 µg/ml), chloramphenicol (0.5, 4, 16, 64, 256, 512 µg/ml), ciprofloxacin (0.5, 4, 16, 64, 256, 512 µg/ml), neomycin (0.5, 4, 16, 64, 256, 512 µg/ml), tetracycline (0.5, 4, 16, 64, 256, 512 µg/ml), ticarcillin (0.5, 4, 16, 64, 256, 512 µg/ml), benzalkonium chloride (3.2, 6.4, 12.8, 25.6, 51.2, 102.4 µg/ml), chlorhexidine (3.2, 6.4, 12.8, 25.6, 51.2, 102.4 µg/ml), and triclosan (0.003, 0.005, 0.01, 0.05, 0.1, 0.5 µg/ml) and number of colonies were scored after incubation at 37°C for 16 h. The percentage of survival for each culture WT, Δ*kpnGH*, Δ*kpnGH*Ω*kpnGH* was monitored by plating approximately 10^5^ cells onto LB agar containing different concentrations of substrates and counting the number of colonies, then calculated *i.e.* number of colony forming units (CFU) on substrate containing plate/number of CFU on LB alone (control)×100. Survival on control plate was 100%. These experiments were performed at least three times.

### Oxidative and Nitrosative Stress Tolerance Assays

The impact of oxidative stress inducing agent (H_2_O_2_) on WT, Δ*kpnGH*, Δ*kpnGH*Ω*kpnGH* was performed using disc assay as reported before [23]. The survivability of cells to oxidative stress was tested by exposing stationary-phase bacteria diluted in LB medium (OD_600 nm_ 0.01) at 37°C to 0.07894 mM, 0.7894 mM, 1.5788 mM, 2.3682 mM and 3.1576 mM for 1 h. The growth kinetics in presence of different nitrosative stress inducing agents [sodium nitroprusside (SNP; concentrations 5 mM, 10 mM, 15 mM, 20 mM, 25 mM) and acidified nitrite; (NaNO2; concentrations 5 mM, 10 mM, 15 mM, 20 mM, 25 mM)] was performed as reported before [23], [24].

### Antibiotic Susceptibility and Determination of Minimum Inhibitory Concentration (MIC)

In this study the strains WT, Δ*kpnGH*, Δ*kpnGH*Ω*kpnGH* were examined for resistance to ampicillin: AMP (10 µg/ml), azithromycin: AZM (15 µg/ml), chloramphenicol: CHL (30 µg/ml), carbencillin: CAR (100 µg/ml), ciprofloxacin: CIP (5 µg/ml), colistin: CST (10 µg/ml), cefepime: CPM (30 µg/ml), ceftriaxone: CTR (30 µg/ml), cephalothin: CTX (10 µg/ml), ceftazidime: CAZ (30 µg/ml), doxycycline: DOX (10 µg/ml), doripenem: DOR (10 µg/ml), ertapenem: ETP (10 µg/ml), linezolid: LZD (10 µg/ml), levofloxacin: LVX (10 µg/ml), oxacillin: OXA (10 µg/ml), polymyxin B: PMB (300 µg/ml), streptomycin: STR (10 µg/ml), tetracycline: TET (30 µg/ml), tigecycline: TGC (10 µg/ml), ticarcillin: TIC (75 µg/ml), tobramycin: TOB (10 µg/ml) and trimethoprim: TMP (5 µg/ml) by using commercial discs (Hi Media, Bombay, India) as described previously according to the interpretation criteria recommended by the Clinical and Laboratory Standards Institute CLSI. *E. coli* ATCC 25922 was used as a reference strain (control) as recommended [23], [24]. MIC of antibiotics was tested using E-strips (Hi Media, Bombay, India). Interpretation was done as per the criteria approved by the CLSI [25].

### Fluorimetric Efflux Studies

Accumulation of EtBr was monitored as described previously [26]. Briefly, bacterial cells were grown to mid-log phase, harvested, and suspended in 1× phosphate buffered saline (0.136 M NaCl, 0.0026 M KCl, 0.01 M Na2HPO4, 0.00176 M KH2PO4; pH 7.0) to an optical density at OD600 nm = 0.2. EtBr was added at a concentration of 10 µg/ml. Aliquots were taken at different time intervals, harvested, suspended in 1 ml of 0.1 M glycine HCl buffer (pH 2.3), and incubated for 6 h at 37°C, and the fluorescence of the supernatant was measured with excitation 530 nm and emission 600 nm. Where indicated, the proton motive force uncoupler CCCP was added to the assay mixture at a final concentration; 25 µg/ml.

### OMP Preparation

OMPs were purified by the method as described previously [23].

### Bioinformatic Analysis and Statistical Analysis

All data are presented as means ± the standard error of the mean. Plotting and calculation of the standard deviation was performed in Microsoft Excel. Statistical analysis was performed on crude data by using a paired Student t test. P values of <0.05 were considered significant.

## Results

### Insilico Analysis of *kpnGH* MFS-type Efflux Pump

The deduced KpnG and KpnH proteins consist of 390 and 511 amino acid residues, respectively, similar to the size of EmrA and EmrB in *E. coli*. The nucleotide sequence deduced from the 2709 bp DNA fragment obtained from *K. pneumoniae* shares >90% identity with EmrAB efflux system in *E. coli*, *S. dysenteriae* and *S.* Typhimurium. Thus, KpnG and KpnH are both members of the MFS family of drug transporters. In addition, significant sequence similarities were detected between KpnGH and EmrAB of *E. coli*, *Proteus vulgaris*, and *Citrobacter freundii*. The multiple alignment of KpnGH with EmrAB of *E. coli* has been shown in Figure S1. The KpnG has two hydrophobic, presumably transmembrane regions spanning from amino acids 23–45; (23 aa in length) and 55–77; (23 aa in length). The KpnF also has thirteen transmembrane regions spanning from amino acids 16–38; (23 aa in length), 52–73; (22 aa in length), 76–98; (23 aa in length), 106–128; (23 aa in length), 138–160; (23 aa in length), 168–188; (21 aa in length), 202–222; (21 aa in length), 230–252; (23 aa in length), 271–2293; (23 aa in length), 307–329; (23 aa in length), 336–358; (23 aa in length), 370–392; (23 aa in length), and 474–496; (23 aa in length). As per SOSUI software average of hydrophobicity was 0.185128 and 0.639921 for *kpnG* and *kpnH* respectively.

### Characterization of *kpnGH* in Hyper Susceptible *E. coli* strain KAM32

The *kpnGH* harbouring cells (*E. coli* KAM32/p*kpnGH*) displayed a elevated resistance to streptomycin (1.5-fold), kanamycin (1.5-fold), erythromycin (4-fold), trimethoprim (2-fold), nalidixic acid (4-fold), SDS (4-fold), bile (4-fold), chlorhexidine (3-fold), benzalkonium chloride (3-fold), and methyl viologen (2-fold) compared to other constructs including *E. coli* KAM32/p*kpnG*, *E. coli* KAM32/p*kpnH*, and *E. coli* KAM32/pUC18 (Table 2).

**Table 2:** Determination of MIC for *E. coli* KAM32/pUC18 and KAM32/p*kpnGH.*

Compounds	KAM32/pUC18	KAM32/p*kpnGH*	Fold change^a^
Erythromycin	4	16	4
Kanamycin	2	4	2
Nalidixic acid	1	4	4
Streptomycin	2	4	2
Trimethoprim	0.125	0.250	2
Benzalkonium chloride	1	3	3
Chlorhexidine	2	6	3
Deoxycholate	1024	8096	4
Methyl viologen	64	128	2
Pyronin Y	4	8	2
Rhodamine 123	8	16	2
SDS	50	200	4

The MIC of various antimicrobial agents such as erythromycin, kanamycin, nalidixic acid, streptomycin, trimethoprim, benzalkonium chloride, chlorhexidine, deoxycholate, methyl viologen, pyronin Y, rhodamine 123, SDS, for *E. coli* (KAM32/pUC18 and KAM32/p*kpnGH*) strains used in this study.

a Fold change is the ratio of MICs for p*kpnGH* to pUC18.

### Contributions of MFS-type Efflux *kpnGH* in *K. pneumoniae* Growth

**Figure 1:**
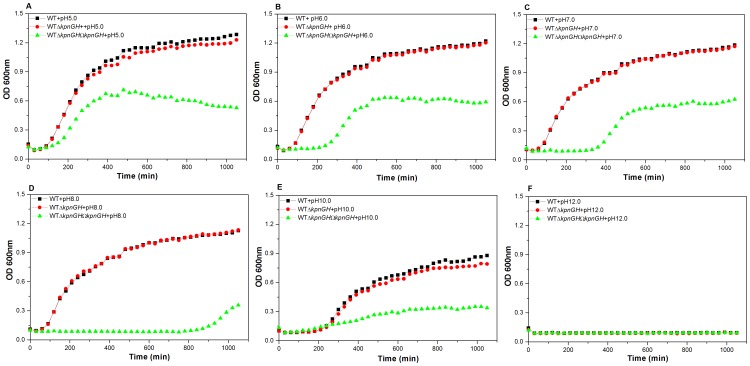
Growth kinetics. Effect on bacterial growth was monitored in WT, Δ*kpnGH* and Δ*kpnGH*Ω*kpnGH* in LB medium at pH 5.0, 6.0, 7.0, 8.0, 10.0 and 12.0. The patterns of representative pH (5.0 (A), 6.0 (B), 7.0 (C), 8.0 (D), 10.0 (E), 12.0 (F)) are shown here. The mutant exhibited 1.782 fold (±0.0002), 2.466 fold (±0.00047), 3.22 fold (±0.000011), 8.46 fold (±0.00058), 1.909 fold (±0.00032), reduced growth compared to WT strain in LB at pH 5.0, 6.0, 7.0, 8.0, and 10.0 respectively. The data presented is the means of triplicate measurements performed three times.

Experimentally the growth characteristics of WT, Δ*kpnGH* (inactivation confirmed by southern and RT-PCR) and Δ*kpnGH*Ω*kpnGH* (confirmed by RT-PCR) were determined over a period of ∼18 h in LB medium with different pH and analysis revealed unique patterns. We tested the growth kinetics at pH 5.0, 6.0, 7.0, 8.0, 10.0 and 12.0 respectively. The mutant exhibited 1.782 fold (±0.0002) reduced growth compared to WT in LB at pH 5.0 [P = 0.0003404] (Figure 1-A). The mutant exhibited 2.466 fold (±0.00047) reduced growth compared to WT in LB at pH 6.0 [P = 0.000337] (Figure 1-B). The mutant exhibited 3.22 fold (±0.000011) reduced growth compared to WT in LB at pH 7.0 [P = 0.000208] (Figure 1-C). The mutant exhibited 8.46 fold (±0.00058) reduced growth compared to WT in LB at pH 8.0 [P = 0.000134] (Figure 1-D). The mutant exhibited 1.909 fold (±0.00032) reduced growth compared to WT in LB at pH 10.0 [P = 0.000164] (Figure 1-E). The other tested pH was 12.0 (Figure 1-F). The motility and biofilm forming ability of *kpnGH* mutant was found to be diminished (Data not shown). Results demonstrate that *kpnGH* influences growth and biofilm forming ability in *K. pneumoniae*.

### Role of Membrane Transporter in Gastrointestinal (GI) Stress Challenges

**Figure 2:**
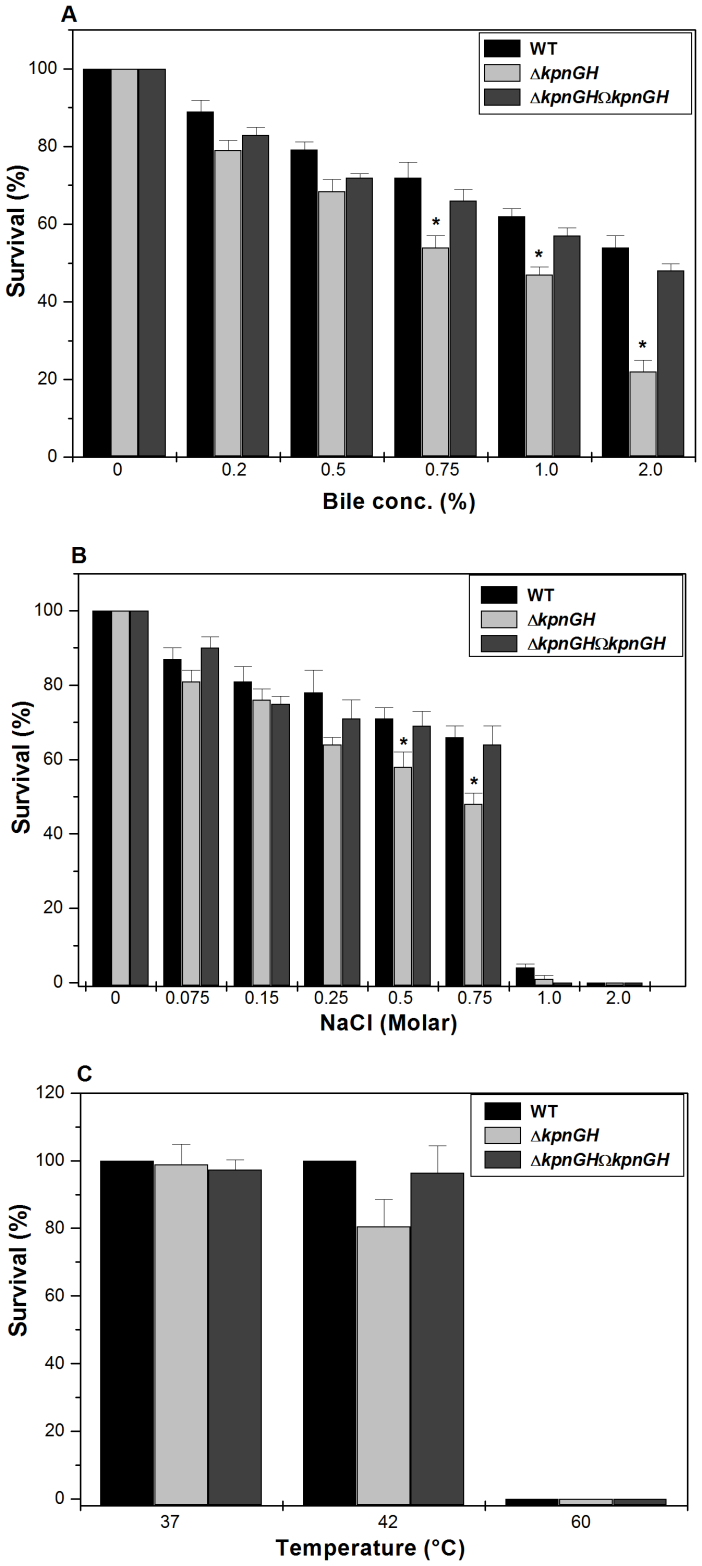
Gastrointestinal stress challenge assays. Survival of wild type, Δ*kpnGH* and Δ*kpnGH*Ω*kpnGH* in different concentrations of A) Bile (0.2%, 0.5%, 0.75%, 1.0%, and 2.0%), B) Osmotic stress (0.075M, 0.15M, 0.25M, 0.5M, 0.75M, 1M and 2M) and C) Different temperatures (37°C, 42°C and 60°C). Percentage survival was calculated by comparing the number of viable cells in LB medium alone. *, Significant difference (P<0.05, Student t test).

To determine the role of *kpnGH* in intestinal colonization, WT, Δ*kpnGH* and Δ*kpnGH*Ω*kpnGH* underwent specific GI stress associated with bile and osmotic challenges. In the bile resistance assay, different strains were exposed to different concentrations (physiological concentration is 0.2% to 2%, [27]) of bile. When mid-log phase cultures were exposed to different concentrations of bile it was observed that the total CFU count of control (measure of surviving capacity) was higher as compared to Δ*kpnGH*. The ability of control to grow in the presence of 0.5% bile was 1.15 fold (±0.042), 0.75% bile was 1.3 fold (±0.024), 1% bile was 1.3 fold (±0.002) and 2% was 2.45 fold (±0.045) higher when compared to Δ*kpnGH*, while transcomplemented Δ*kpnGH*Ω*kpnGH* strain restored the ability to tolerate stress (Figure 2-A) [P = 0.02157191].

The ability of control to grow in the presence of NaCl (physiological concentration being 150 mM, [28]) at 0.25M was ∼1.218 fold (±0.033), 0.5M was ∼1.23 fold (±0.015), and 0.75M was ∼1.375 fold (±0.14), higher when compared to Δ*kpnGH* regardless of the inoculum size (Figure 2-B) [P = 0.018152256].

To deduce the role of *kpnGH* in temperature tolerance, we performed the heat shock assay. The temperature dependent assay showed that the *kpnGH* mutant displayed 15% reduced survival than the wild type at 42°C, thereby demonstrating that the response of *K. pneumoniae kpnGH* mutant in temperature stress (Figure 2-C). Overall these results imply that *kpnGH* had a contributory role towards varied stress tolerance in *K. pneumoniae*.

### Role of *kpnGH* in Oxidative Stress Response

To deduce the role of *kpnGH* in oxidative stress, we performed the hydrogen peroxide challenge assay. Disc diffusion assay showed that the *kpnGH* mutant exhibited 1.07 fold greater sensitivity to 30% H_2_O_2_ (inhibition zone = 42±1.0 mm) than the wild-type (inhibition zone = 39±1.5 mm) (Figure S2-A) [P = 0.225], clearly demonstrating the role of *K. pneumoniae kpnGH* in oxidative stress.

The sensitivity of stationary-phase cultures to oxidative stress was tested by exposing them to a range of H_2_O_2_ concentrations (0.07894 mM, 0.7894 mM, 1.5788 mM, 2.3682 mM to 3.1576 mM) for 1 h. Only 42% and 17% of the Δ*kpnGH* cells survived upon treating with 0.07894 mM, 0.7894 mM of hydrogen peroxide as compared to the 91% and 77% survival observed in control cells respectively (Figure S2-B).

### Involvement of *kpnGH* in Nitrosative Stress Response

SNP is a nitrosative stress inducer. It was interesting to note that *kpnGH* mutant exhibited 3.024-fold (±0.032; P = 0.000216), 3.38-fold (±0.072; P = 0.000241), 2.58-fold (±0.066; P = 0.000232), 1.702-fold (±0.012; P = 0.000141), 2.194-fold (±0.054; P = 0.000581), 1.132-fold (±0.076; P = 0.0002814), reduced growth compared to WT strain in LB at 0 mM, 5 mM, 10 mM, 15 mM, 20 mM and 25 mM respectively (Figure S2, C I–IV).

To further evaluate the potentiality of *K. pneumoniae kpnGH* against other reactive nitrogen species, we tested tolerance of *kpnGH* inactivated cells towards acidified sodium nitrite. Protonated nitrite quickly degrades to generate numerous species of nitrogen oxides for example nitric oxide [27]. It was interesting to note that *kpnGH* mutant exhibited 2.88-fold (±0.045; P = 0.000115), 1.838-fold (±0.087; P = 0.000316), 1.629-fold (±0.026; P = 0.000211), 1.643-fold (±0.097; P = 0.000510), 1.454-fold (±0.032; P = 0.000409), 1.602-fold (±0.74; P = 0.000311), reduced growth compared to WT strain in LB at 0 mM, 5 mM, 10 mM, 15 mM, 20 mM and 25 mM respectively (Figure S2, D I–IV). Overall, these results imply that *kpnGH* had a role towards nitrosative stress tolerance in *K. pneumoniae*.

### Functions of KpnGH in Antibiotic Resistance

**Figure 3:**
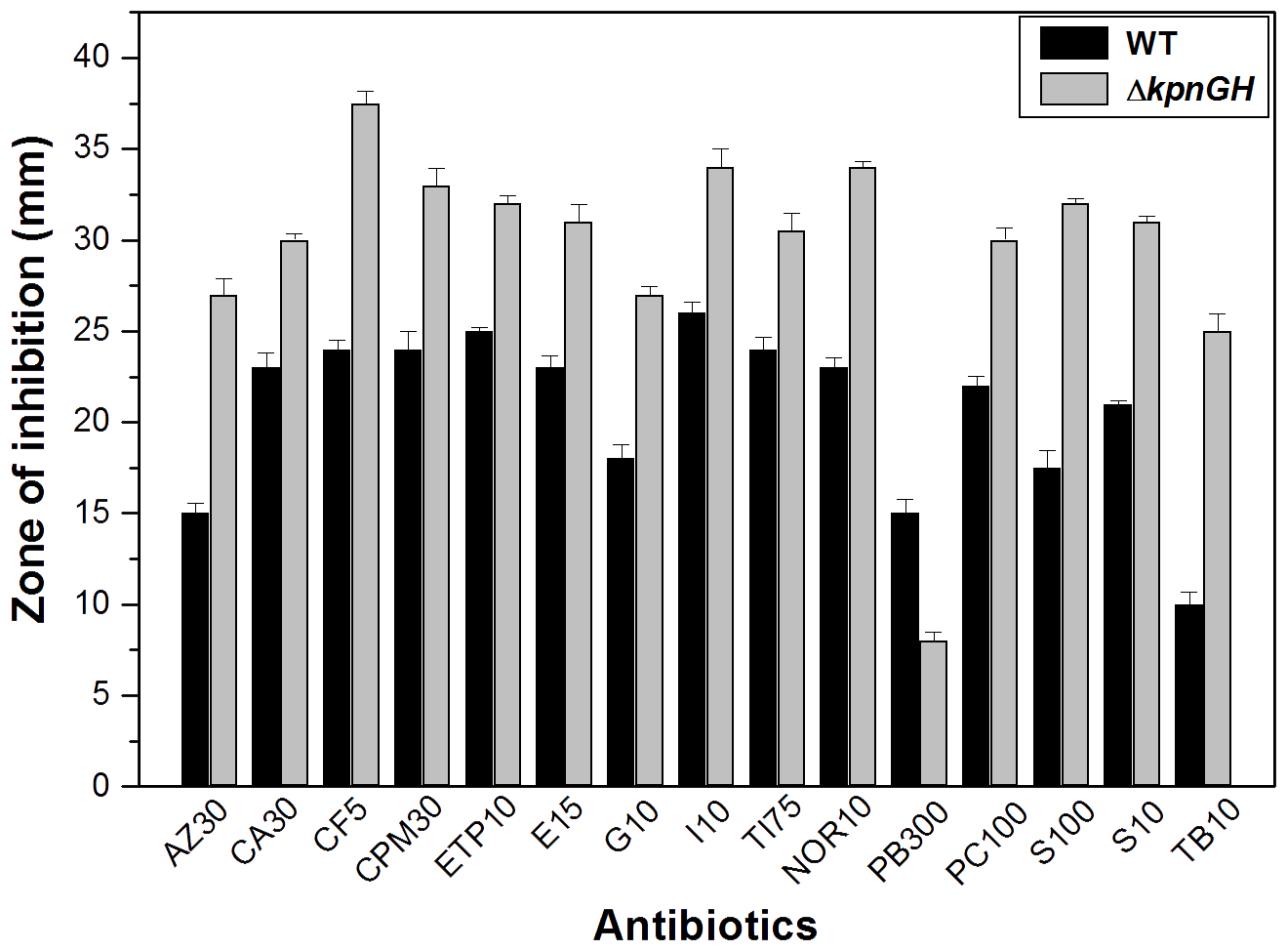
Antimicrobial Susceptibility assays. The Kirby Bauer disc diffusion assay was performed with different antibiotics using commercial discs. Data for representative drugs have been shown here.

To evaluate the role of *kpnGH* in drug resistance, antibiotic susceptibilities of WT and Δ*kpnGH* was monitored. The results of disc diffusion assay displayed that upon inactivation of the efflux pump, the bacterial cells displayed significantly altered susceptibility to azithromycin, ceftazidime, ciprofloxacin, ertapenem, erythromycin, gentamicin, imipenem, ticarcillin, norfloxacin, polymyxin-B, piperacillin, spectinomycin, tobramycin and streptomycin (Figure 3).

The precise minimum inhibitory concentration (MIC) was further evaluated by following the guidelines of CLSI by E-test [25]. The MIC of *K. pneumoniae* for different antibiotics namely was, ceftazidime 0.256 µg/ml, cefepime 2.56 µg/ml, ceftriaxone 2.56 µg/ml, nalidixic acid 0.1 µg/ml, tobramycin 0.1 µg/ml, streptomycin 0.1 µg/ml, spectinomycin 0.1 µg/ml, tetracycline 5 µg/ml whereas MIC for Δ*kpnGH* {fold increase in brackets} for the same line of drugs were ceftazidime 0.064 µg/ml {4 fold}, cefepime 0.64 µg/ml {4 fold}, ceftriaxone 0.64 µg/ml {4 fold}, nalidixic acid 0.05 µg/ml {2 fold}, tobramycin 0.05 µg/ml {2 fold}, streptomycin 0.01 µg/ml {10 fold}, spectinomycin 0.01 µg/ml {10 fold}, tetracycline 1 µg/ml {5 fold} (Table 3). Complementation restored the defective phenotype. Overall the data indicates that MFS-efflux pump has multiple substrates.

**Table 3:** Determination of MIC for WT (*K. pneumoniae*), Δ*kpnGH* and Δ*kpnGH*Ω*kpnGH.*

Antibiotics	WT (µg/ml)	Δ*kpnGH* (µg/ml)	Fold change^a^	Δ*kpnGH*Ω*kpnGH*
Cefepime	2.56	0.64	4	2.56
Ceftazidime	0.256	0.064	4	0.256
Ceftriaxone	2.56	0.64	4	2.56
Ciprofloxacin	<0.01	<0.005	2	<0.01
Erythromycin	>4	2	2	4
Spectinomycin	0.1	0.01	10	0.1
Streptomycin	0.1	0.01	10	0.1
Tetracycline	5	1	5	5
Tobramycin	0.1	0.05	2	0.1

a Fold change is the ratio of MICs for WT to Δ*kpnGH.*

### Mutation in *kpnGH* Increases Sensitivity to Structurally Unrelated Compounds

The WT, Δ*kpnGH* and Δ*kpnGH*Ω*kpnGH* cultures in this study were tested for their ability to withstand high concentration of different substrates that are structurally unrelated to antibiotics. The ability of Δ*kpnGH* to withstand different concentrations of EtBr (figure S3-A) and acriflavine (figure S4-B) was reduced by 1.3 and 2.0 fold when compared to WT respectively. Results were similar when done with other dyes such as rhodamine, safranine and acridine orange (data not shown).

Upon exposing the cells to different concentrations of SDS it was observed that the total CFU count of WT at 16384 µg/ml was 1.5 fold higher than Δ*kpnGH* [*P* = /0.009962331] (figure S3-C). Analysis indicates the wide substrate specificity for *kpnGH* in *K. pneumoniae*.

The growth rate of Δ*kpnGH* in the presence of 2.0 µg/ml EtBr, was >12 fold lesser when compared to that of WT (P = 0.000477). To distinguish whether this is due to the loss of efflux pump activity in *kpnGH* mutant, the growth profile was monitored with CCCP, an uncoupler known to collapse the membrane energy and block the energy-dependent efflux pump. As expected a substantial decrease in growth was observed in *kpnGH* mutant (15.18-fold; P = 0.000697), (1.39-fold; P = 0.000775), (6.32-fold; P = 0.000681) and (15.06-fold; P = 0.000174) in the presence of CCCP, reserpine, verampamil and 2, 4 DNP respectively (Figure S3, D I–IV).

The growth rate of Δ*kpnGH* in the presence of 2.0 µg/ml rhodamine, was >17.5 fold lesser when compared to that of WT (P = 0.000153). Akin a substantial decrease in growth was observed in *kpnGH* mutant (10.476-fold; P = 0.000234), (1.39102-fold; P = 0.000796), (8.13-fold; P = 0.000101) and (23.1-fold; P = 0.000196) in the presence of CCCP, reserpine, verampamil and 2, 4 DNP respectively (Figure S3, E I–IV).

The growth rate of Δ*kpnGH* in the presence of 2.0 µg/ml safranine, was >15.94 fold lesser when compared to that of WT (P = 0.000221). A decrease in growth was observed in *kpnGH* mutant (5.16-fold; P = 0.000251), (0.988-fold; P = 0.000182), (16.23-fold; P = 0.000215) and (12.5-fold; P = 0.000101) in the presence of CCCP, reserpine, verampamil and 2, 4 DNP respectively (Figure S3, F I–IV). The mutant lacks *kpnGH*, the efflux pump in its functional form, so possibly leading to decreased efflux activity and thereby we observe a stunted growth profile.

### Tolerance to Different Classes of Antibiotics

**Figure 4:**
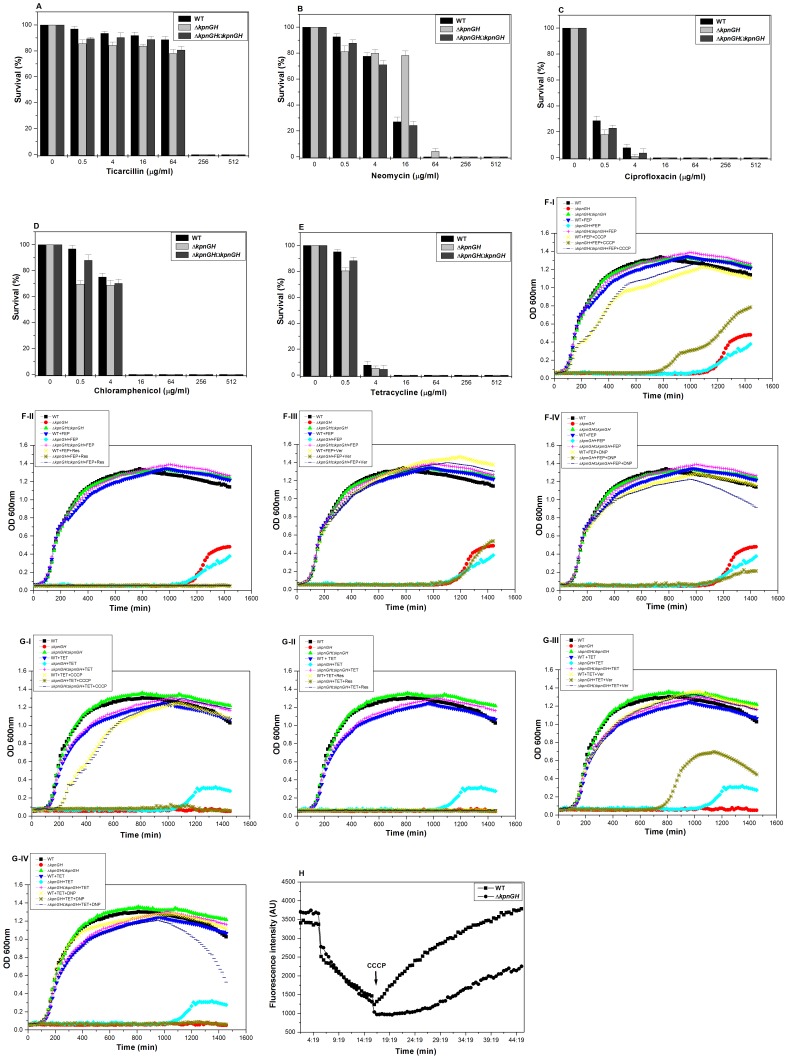
Susceptibility, Growth inactivation and accumulation assays towards different antibiotics. A) Sensitivity of WT, Δ*kpnGH* and Δ*kpnGH*Ω*kpnGH* towards ticarcillin. Upon exposing the cells to different concentrations of ticarcillin it was observed that the total CFU count of WT at 0.5 µg/ml was 1 fold, 4 µg/ml was 1.13 fold, 16 µg/ml was 1.11 fold, 64 µg/ml was 1.09 fold, 256 µg/ml was 1.09 fold, 512 µg/ml was 1.13 fold increased than Δ*kpnGH* [*P* = 0.027]. The data is the means of measurements made in triplicate and performed three times. B) Sensitivity of WT, Δ*kpnGH* and Δ*kpnGH*Ω*kpnGH* towards neomycin. Upon exposing the cells to different concentrations of neomycin it was observed that the total CFU count of WT at 0.5 µg/ml was 1.14 fold, 4 µg/ml was 0.97 fold, 16 µg/ml was 0.34 fold increased than Δ*kpnGH* [*P* = 0.042]. The data is the means of measurements made in triplicate and performed three times. C) Sensitivity of WT, Δ*kpnGH* and Δ*kpnGH*Ω*kpnGH* towards ciprofloxacin. Exposing the cells to different concentrations of ciprofloxacin it was observed that the total CFU count of WT at 0.5 µg/ml was 1.5 fold, 4 µg/ml was 7.3 fold increased than Δ*kpnGH* [*P* = 0.0188]. The data is the means of measurements made in triplicate and performed three times. D) Sensitivity of WT, Δ*kpnGH* and Δ*kpnGH*Ω*kpnGH* towards chloramphenicol. Exposing the cells to different concentrations of chloramphenicol it was observed that the total CFU count of WT at 0.5 µg/ml was 1.39 fold, 4 µg/ml was 1.08 fold increased than Δ*kpnGH* [*P* = 0.0266]. The data is the means of measurements made in triplicate and performed three times. E) Sensitivity of WT, Δ*kpnGH* and Δ*kpnGH*Ω*kpnGH* towards tetracycline. Exposing the cells to different concentrations of tetracycline it was observed that the total CFU count of WT at 0.5 µg/ml was 1.18 fold, 4 µg/ml was 1.45 fold increased than Δ*kpnGH* [*P* = 0.0288]. The data is the means of measurements made in triplicate and performed three times. F) Growth inactivation assay using wild type, Δ*kpnGH* and Δ*kpnGH*Ω*kpnGH* in the presence of cefepime. Growth pattern in absence of any drug or inhibitor is included as control. The growth rate of Δ*kpnGH* in the presence of 0.1 µg/ml cefepime, was 15.373 fold lesser when compared to that of WT (P = 0.000574). A decrease in growth was observed in *kpnGH* mutant (7.474-fold; P = 0.000123), (1.032-fold; P = 0.000159), (16.23-fold; P = 0.000215) and (12.5-fold; P = 0.000101) in the presence of CCCP (I), reserpine (II), verampamil (III) and 2, 4 DNP (IV) respectively. The mean values of three independent experiments have been used for plotting the graph. G) Growth inactivation assay using wild type, Δ*kpnGH* and Δ*kpnGH*Ω*kpnGH* in the presence of tetracycline. Growth pattern in absence of any drug or inhibitor is included as control. The growth rate of Δ*kpnGH* in the presence of 0.01 µg/ml tetracycline, was 10.655 fold lesser when compared to that of WT (P = 0.000234). A decrease in growth was observed in *kpnGH* mutant (10.476-fold; P = 0.000477), (1.102-fold; P = 0.000796), (8.134-fold; P = 0.000101) (15.28-fold; P = 0.000101) in the presence of CCCP (I), reserpine (II), verampamil (III) and 2, 4 DNP (IV) respectively. The mean values of three independent experiments have been used for plotting the graph. H) Accumulation studies using EtBr with control strain and Δ*kpnGH*. Efflux of EtBr in mutant and wild type cells was monitored continuously by measuring fluorescence emission at 600 nm upon excitation at 530 nm. After 5 mins in flourimeter, cells loaded with EtBr were energized by addition of glucose and efflux of EtBr was monitored. After 10 mins, 100 µM CCCP was added as indicated to abolish active transport and fluorescence emission was monitored further. The fluorescence was measured using spectrofluorometer (Hitachi). Each data point represents the mean plus the standard deviation of three independent experiments.

Upon exposing the cells to different concentrations of ticarcillin it was observed that the total CFU count of WT at 0.5 µg/ml was 1 fold, 4 µg/ml was 1.13 fold, 16 µg/ml was 1.11 fold, 64 µg/ml was 1.09 fold, 256 µg/ml was 1.09 fold, 512 µg/ml was 1.13 fold increased than Δ*kpnGH* [*P* = 0.027] (Figure 4-A).

Upon exposing the cells to different concentrations of neomycin it was observed that the total CFU count of WT at 0.5 µg/ml was 1.14 fold, 4 µg/ml was 0.97 fold, 16 µg/ml was 0.34 fold increased than Δ*kpnGH* [*P* = 0.042] (Figure 4-B).

Exposing the cells to different concentrations of ciprofloxacin it was observed that the total CFU count of WT at 0.5 µg/ml was 1.5 fold, 4 µg/ml was 7.3 fold increased than Δ*kpnGH* [*P* = 0.0188] (Figure 4-C).

Exposing the cells to different concentrations of chloramphenicol it was observed that the total CFU count of WT at 0.5 µg/ml was 1.39 fold, 4 µg/ml was 1.08 fold increased than Δ*kpnGH* [*P* = 0.0266] (Figure 4-D).

Exposing the cells to different concentrations of tetracycline it was observed that the total CFU count of WT at 0.5 µg/ml was 1.18 fold, 4 µg/ml was 1.45 fold increased than Δ*kpnGH* [*P* = 0.0288] (Figure 4-E). Results indicate the role of KpnGH in drug resistance.

The growth rate of Δ*kpnGH* in the presence of 0.1 µg/ml cefepime, was 15.373 fold lesser when compared to that of WT (P = 0.000574). A decrease in growth was observed in *kpnGH* mutant (7.474-fold; P = 0.000123), (1.032-fold; P = 0.000159), (16.23-fold; P = 0.000215) and (13.7-fold; P = 0.000176) in the presence of CCCP, reserpine, verampamil and 2, 4 DNP respectively (Figure 4, F I–IV).

The growth rate of Δ*kpnGH* in the presence of 0.01 µg/ml tetracycline, was 10.655 fold lesser when compared to that of WT (P = 0.000234). A decrease in growth was observed in *kpnGH* mutant (10.476-fold; P = 0.000477), (1.102-fold; P = 0.000796), (8.134-fold; P = 0.000101) and (15.28-fold; P = 0.000101) in the presence of CCCP, reserpine, verampamil and 2, 4 DNP respectively (Figure 4, G I–IV).

The EtBr accumulation data indicate that the efflux was most efficient in the control, whereas it was less efficient in Δ*kpnGH*. The addition of CCCP increased the EtBr levels which eventually reached a plateau in both control and Δ*kpnGH* (Figure 4-H). Overall, these preliminary results suggest that mutant of *kpnGH* possibly affects active efflux in *K. pneumoniae.*

### MFS Efflux Pump *kpnGH* Influences Tolerance to Disinfectants

We tested the susceptibilities of control and Δ*kpnGH* towards different concentrations of popularly used hospital based disinfectants such as chlorhexidine and benzalkonium chloride [29], [30]. Upon exposing the cells to different concentrations of benzalkonium chloride it was observed that the total CFU count of control was 5 to 12 fold higher than Δ*kpnGH* [*P = *0.047277226] (figure S4-A).

When cells were exposed to different concentrations of chlorhexidine it was observed that the total CFU count of control was 1.3 to 1.7 fold higher than Δ*kpnGH* [*P* = /0.080120359] (figure S4-B).

Upon exposing the cells to different concentrations of triclosan it was observed that the total CFU count of control was 1.3 to 1.4 fold higher than Δ*kpnGH* [*P* = /0.065323347] (figure S4-C). Decisively, the results imply the broad spectrum antimicrobial resistance property of *kpnGH*, a MFS-type efflux pump for the first time in *K. pneumoniae.*

### Alterations in Outer Membrane Profile of the *kpnGH* Inactivated Mutant in *K. pneumoniae*

A reduction in the permeation of antibiotics is generally related to a decrease in porin expression or an alteration in the porin structure [2], [5], [6]. Thus, we compared the OMP profiles of Δ*kpnGH* with WT to find out whether a *kpnGH* inactivated mutant expresses alternative porins/OMPs to maintain normal cellular functions. It was interesting to note that there was a marked difference in the OMP profiles of mutant compared to wild type (Figure S5), and deciphering the identity and function of these proteins is highly warranted.

## Discussion

*Klebsiella pneumoniae,* a member of Enterobacteriaceae family, is an important pathogen in both the community and the clinical setting. *K. pneumoniae* is largely responsible for causing diseases such as nosocomial pneumonia (7 to 14% of all cases), septicaemia (4 to 15% of all cases), wound infections (2 to 4% of all cases), and neonatal septicaemia (3 to 30% of all cases) [1]. Few reports have assessed the masked role of efflux pumps in multidrug resistant phenotype in *K. pneumoniae* clinical isolates. Analysis of *K. pneumoniae* genome sequences namely *K. pneumoniae* strain 1084 (NC_018522.1); *K. pneumoniae* strain HS11286 (NC_016845.1); *K. pneumoniae* strain MGH 78578 (NC_009648.1); as well as *K. pneumoniae* strain NTUH-K2044 (NC_012731.1) indicates the presence of several putative multidrug efflux pumps [10]. In this study, we report the biological functions of MFS-type efflux pump *kpnGH* in modulating the cellular physiology and antimicrobial resistance in *K. pneumoniae* for the first time.

The *E. coli* KAM32 cells expressing *kpnGH* mounted significant growth difference than the vector control on a broad range of antimicrobial compounds, including representative antimicrobial agents, antiseptics, cationic dyes and detergents confirming that *K. pneumoniae kpnGH,* like the *emrAB* homolog in *E. coli, S.* Typhimurium, is required for resistance to various antimicrobials [21], [31], [32]. While inactivation of *kpnEF* resulted in increased susceptibility towards cefepime, ceftriaxone, imipenem, ertapenem, enrofloxacin, norfloxacin and ciprofloxacin, however non functional *kpnGH* results in increased susceptibility to cefepime, ceftazidime, ceftriaxone, ciprofloxacin, erythromycin, spectinomycin, streptomycin, tetracycline and tobramycin and structurally unrelated compounds such as SDS, deoxycholate, EtBr, and acriflavine. KpnGH displayed sensitivity to benzalkonium chloride, chlorhexidine and triclosan; however KpnEF displayed greater sensitivity to these compounds. Results presented here provide evidence that inactivation of *kpnGH* diminishes drug efflux capacity in *K. pneumoniae*.

Where the previously reported efflux pump *kpnEF* displayed higher sensitivity to high bile (∼4.0 fold) concentration, interestingly, the *kpnGH* mutant is only 2.5 fold sensitive to varied bile challenges indicating that *K. pneumoniae* possibly utilizes *kpnGH* to extrude bile salts from the cytoplasm out of the cell to successfully thrive in the GI tract of human host [23]. Evidence indicating the role of KpnGH in surviving conditions that mimic the upper parts of the GI, where they encounter hyper osmotic condition in a microaerobic environment adds to the multifaceted, unprecedented functions displayed by MFS-type efflux pump in *K. pneumoniae.* In conclusion, using various molecular approaches, we have demonstrated the functions of *kpnGH*, a MFS-type efflux pump in cellular physiology and antimicrobial resistance in *Klebsiella* spp for the first time.

## Supporting Information

Figure S1 Sequence alignment of KpnGH with EmrAB homolog. Sequence alignments of *Klebsiella pneumoniae* EmrA (KP1_4279) with *Escherichia coli* EmrA EmrA (b2685) were made in CLUSTAL Omega (https://www.ebi.ac.uk/Tools/msa/clustalo). The conserved domain analysis (http://www.ncbi.nlm.nih.gov/Structure/cdd/wrpsb.cgi) revealed the Biotin_lipoyl_2 domain (pfam 13533) and HlyD family secretion protein domain (pfam 13437) are highlighted in yellow and cyan color respectively. The asterisks indicate fully conserved residues, colons strongly similar residues, dots weakly similar residues.
(DOCX)

Figure S2 Oxidative and nitrosative challenge assays. A) The ability of wild type, Δ*kpnGH* and Δ*kpnGH*Ω*kpnGH* to combat different levels of hydrogen peroxide was measured by disc diffusion assay. B) Survival of wild type, Δ*kpnGH* and Δ*kpnGH*Ω*kpnGH* strains monitored upon exposure to H_2_O_2_ at 0.07894 mM, 0.7894 mM, 1.5788 mM, 2.3682 mM and 3.1576 mM. After 1 h of treatment with 0.07894 mM hydrogen peroxide, only 42% of Δ*kpnGH* cells survived in comparison to 91% of the wild-type cells. C) Growth pattern of wild type, Δ*kpnGH* and Δ*kpnGH*Ω*kpnGH* in the presence of SNP. The growth kinetics of *kpnGH* exhibited 3.024-fold (±0.032; P = 0.000216), 3.38-fold (±0.072; P = 0.000241), 2.58-fold (±0.066; P = 0.000232), 1.702-fold (±0.012; P = 0.000141), 2.194-fold (±0.054; P = 0.000581), 1.132-fold (±0.076; P = 0.0002814) reduced growth compared to WT strain in LB at 0 mM, 5 mM, 10 mM, 15 mM, 20 mM and 25 mM respectively. The mean values of three independent experiments have been used for plotting the graph. D) Growth pattern of wild type, Δ*kpnGH* and Δ*kpnGH*Ω*kpnGH* in the presence of NaNO2. The growth kinetics of *kpnGH* exhibited 2.88-fold (±0.045; P = 0.000115), 1.838-fold (±0.087; P = 0.000316), 1.629-fold (±0.026; P = 0.000211), 1.643-fold (±0.097; P = 0.000510), 1.454-fold (±0.032; P = 0.000409), 1.602-fold (±0.74; P = 0.000311) reduced growth compared to WT strain in LB at 0 mM, 5 mM, 10 mM, 15 mM, 20 mM and 25 mM respectively. The mean values of three independent experiments have been used for plotting the graph.
(TIF)

Figure S3 Susceptibility and Growth inactivation assays towards different dyes and detergent. A) Sensitivity of wild type, Δ*kpnGH* and Δ*kpnGH*Ω*kpnGH* towards EtBr when cells were exposed to different concentrations of dye (2 µg/ml, 4 µg/ml, 64 µg/ml, 128 µg/ml, 256 µg/ml and 512 µg/ml). The data is the means of measurements made in triplicate and performed three times. B) Sensitivity of wild type, Δ*kpnGH* and Δ*kpnGH*Ω*kpnGH* towards acriflavine when cells were exposed to different concentrations of dye (2 µg/ml, 4 µg/ml, 64 µg/ml, 128 µg/ml, 256 µg/ml and 512 µg/ml). The data is the means of measurements made in triplicate and performed three times. C) Sensitivity of wild type, Δ*kpnGH* and Δ*kpnGH*Ω*kpnGH* towards SDS when cells were exposed to different concentrations of detergent (1024 µg/ml, 2048 µg/ml, 4096 µg/ml, 8192 µg/ml, 16834 µg/ml). The data is the means of measurements made in triplicate and performed three times. D) Growth inactivation assay using wild type, Δ*kpnGH* and Δ*kpnGH*Ω*kpnGH* in the presence of EtBr. Growth pattern in absence of any EtBr or inhibitor is included as control. The growth rate of Δ*kpnGH* in the presence of 2.0 µg/ml EtBr, was >12 fold lesser when compared to that of WT (P = 0.000477). A decrease in growth was observed in *kpnGH* mutant (15.18-fold; P = 0.000697), (1.39-fold; P = 0.000775), (6.32-fold; P = 0.000681) and (15.06-fold; P = 0.000174) in the presence of CCCP (I), reserpine (II), verampamil (III) and 2, 4 DNP (IV) respectively. The mean values of three independent experiments have been used for plotting the graph. E) Growth inactivation assay using wild type, Δ*kpnGH* and Δ*kpnGH*Ω*kpnGH* in the presence of rhodamine. Growth pattern in absence of any rhodamine or inhibitor is included as control. The growth rate of Δ*kpnGH* in the presence of 2.0 µg/ml rhodamine, was >17.5 fold lesser when compared to that of WT (P = 0.000153). A decrease in growth was observed in *kpnGH* mutant (10.476-fold; P = 0.000234), (1.39102-fold; P = 0.000796), (8.13-fold; P = 0.000101) and (23.1-fold; P = 0.000196) in the presence of CCCP (I), reserpine (II), verampamil (III) and 2, 4 DNP (IV) respectively. The mean values of three independent experiments have been used for plotting the graph. F) Growth inactivation assay using wild type, Δ*kpnGH* and Δ*kpnGH*Ω*kpnGH* in the presence of safranine. Growth pattern in absence of any safranine or inhibitor is included as control. The growth rate of Δ*kpnGH* in the presence of 2.0 µg/ml safranine, was >15.94 fold lesser when compared to that of WT (P = 0.000221). A decrease in growth was observed in *kpnGH* mutant (5.16-fold; P = 0.000251), (0.988-fold; P = 0.000182), (16.23-fold; P = 0.000215) and (12.5-fold; P = 0.000101) in the presence of CCCP (I), reserpine (II), verampamil (III) and 2, 4 DNP (IV) respectively. The mean values of three independent experiments have been used for plotting the graph.
(TIF)

Figure S4 Disinfectant challenge assays. Survival of wild type, Δ*kpnGH* and Δ*kpnGH*Ω*kpnGH* in the presence of different concentrations (µg/ml) of A) benzalkonium chloride (3.2, 6.4, 12.8, 25.6, 51.2, 102.4), B) chlorhexidine (3.2, 6.4, 12.8, 25.6, 51.2, 102.4), C) triclosan (0.003, 0.005, 0.01, 0.05, 0.1, 0.5). The percent survival was calculated by comparison to the numbers of viable cells obtained in LB medium alone. *, Significant difference (P<0.05, Student t test).
(TIF)

Figure S5 Protein profiling of WT and *kpnGH* mutants. Membrane protein profiles were compared between the WT strain, and *kpnGH* mutant. About 20 µg of total protein lysate was loaded in an order as follows; lane 2: WT, lane 3: *kpnGH* mutant 1, lane 4: *kpnGH* mutant 2, lane 5: *kpnGH* mutant3, lane 6: *kpnGH* mutant 4, lane 7: *kpnGH* mutant 5 respectively. A similar amount of outer membrane fractions was loaded in an order as follows; lane 8: WT, lane 9: *kpnGH* mutant 1, lane 10: *kpnGH* mutant 2, lane 11: *kpnGH* mutant3, lane 12: *kpnGH* mutant 4, lane 13: *kpnGH* mutant 5 respectively. The inner membrane fractions was loaded in an order as follows; lane 14: WT, lane 15: *kpnGH* mutant 1, lane 16: *kpnGH* mutant 2, lane 17: *kpnGH* mutant3, lane 18: *kpnGH* mutant 4, lane 19: *kpnGH* mutant 5 respectively. Equal protein concentrations were separated by SDS-PAGE with a 5% stacking gel and a 12% separating gel and stained with coomassie brilliant blue. Lane M has molecular weight standards.
(TIF)
