# Supplementary material for: Role of Novel Multidrug Efflux Pump Involved in Drug Resistance in Klebsiella pneumoniae
Source: PLoS One. 2014 May 13;9(5):e96288. doi: 10.1371/journal.pone.0096288 (PMC4019481; doi:10.1371/journal.pone.0096288)
Supplement: Figure S1 — Sequence alignment of KpnGH with EmrAB homolog. Sequence alignments of Klebsiella pneumoniae EmrA (KP1_4279) with Escherichia coli EmrA EmrA (b2685) were made in CLUSTAL Omega (https://www.ebi.ac.uk/Tools/msa/clustalo). The conserved domain analysis (http://www.ncbi.nlm.nih.gov/Structure/cdd/wrpsb.cgi) revealed the Biotin_lipoyl_2 domain (pfam 13533) and HlyD family secretion protein domain (pfam 13437) are highlighted in yellow and cyan color respectively. The asterisks indicate fully conserved residues, colons strongly similar residues, dots weakly similar residues. (DOCX) [file pone.0096288.s001.docx]

**Figure S1**

**CLUSTAL O (1.2.1) Multiple sequence alignment**

KP1_4279 MSANAESQTPQQPGSKKGKRKGALLLLTLLFIIIAVAYGIYWFLVLRHYEETDDAYVAGN

b2685 MSANAETQTPQQPVKKSGKRKRLLLLLTLLFIIIAVAIGIYWFLVLRHFEETDDAYVAGN

******:****** .*.**** ************** **********:***********

KP1_4279 QVQIMAQVAGSVTKVWADNTDYVQKGDPLVTLDRTDAQQAFEKAKTQLAASVRQTRQQMI

b2685 QIQIMSQVSGSVTKVWADNTDFVKEGDVLVTLDPTDARQAFEKAKTALASSVRQTHQLMI

*:***:**:************:*::** ***** ***:******** **:*****:* **

KP1_4279 NSKQLQANIDVKKTALAQAQADLNRRIPLGAANLIGREELQHARDTVASAQAELDVAIQQ

b2685 NSKQLQANIEVQKIALAKAQSDYNRRVPLGNANLIGREELQHARDAVTSAQAQLDVAIQQ

*********:*:* ***:**:* ***:*** **************:*:****:*******

KP1_4279 YNANQAIVLGTRLEQQPAVLQAATEVRNAWLALQRTQIVSPISGYVSRRSVQPGAQIGTT

b2685 YNANQAMILGTKLEDQPAVQQAATEVRNAWLALERTRIISPMTGYVSRRAVQPGAQISPT

******::***:**:**** *************:**:*:**::******:*******. *

KP1_4279 TPLMAVVPATNLWIDANFKETQLAHMRIGQPATVISDIYGDDVKYTGKVVGLDMGTGSAF

b2685 TPLMAVVPATNMWVDANFKETQIANMRIGQPVTITTDIYGDDVKYTGKVVGLDMGTGSAF

***********:*:********:*.******.*: :************************

KP1_4279 SLLPAQNATGNWIKVVQRLPVRIELDEKQLAEHPLRIGLSTLVEVNTTDRDGEMLASQVR

b2685 SLLPAQNATGNWIKVVQRLPVRIELDQKQLEQYPLRIGLSTLVSVNTTNRDGQVLANKVR

**************************:*** ::**********.****:***::**.:**

KP1_4279 SSPVYESNAREIALDPVNKLIDEIIQANAG

b2685 STPVAVSTAREISLAPVNKLIDDIVKANAG

*:** *.****:* *******:*::****
