# Supplementary figures and images for: Role of Novel Multidrug Efflux Pump Involved in Drug Resistance in Klebsiella pneumoniae
Source: PLoS One. 2014 May 13;9(5):e96288. doi: 10.1371/journal.pone.0096288 (PMC4019481; doi:10.1371/journal.pone.0096288)

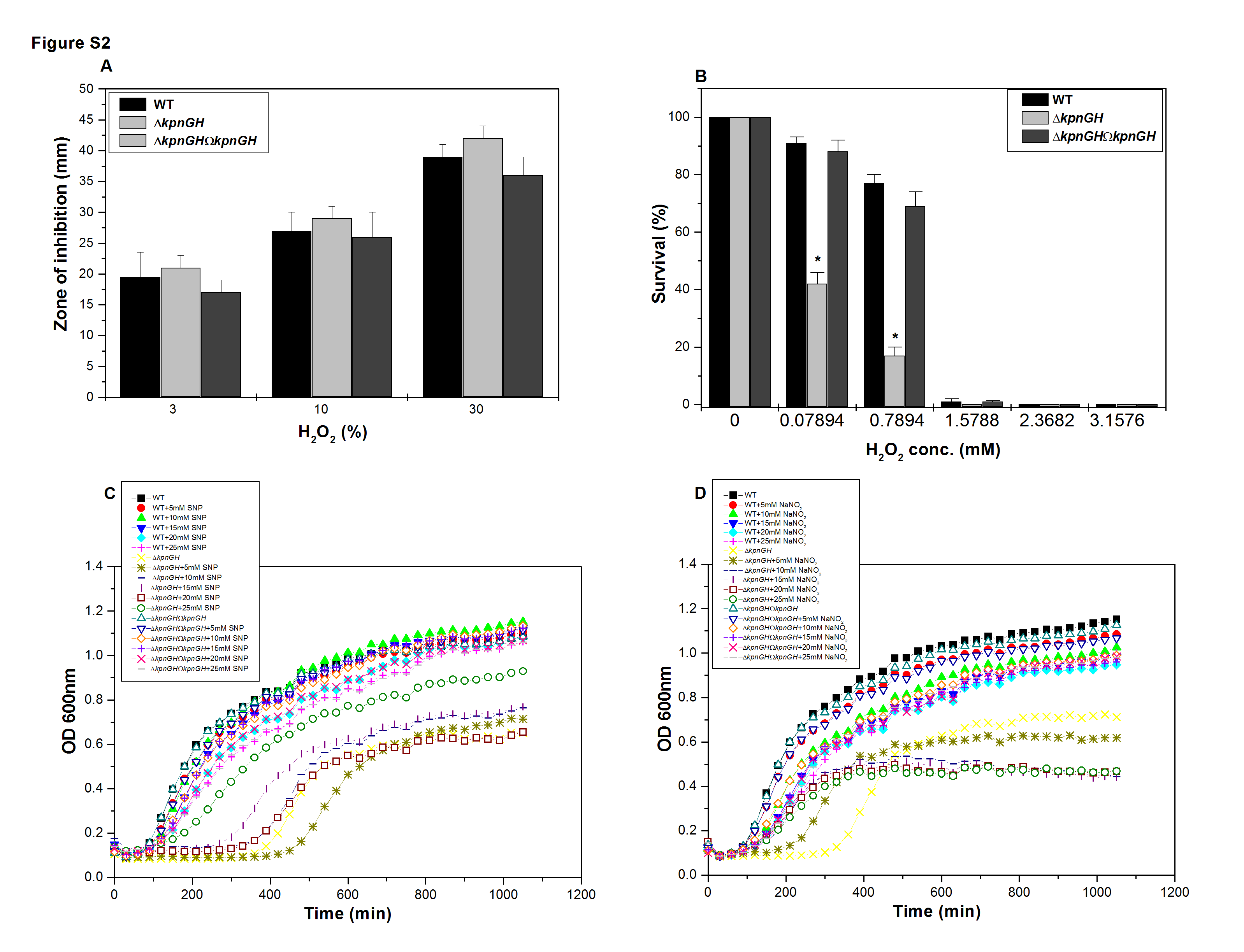

Supplement: Figure S2 — Oxidative and nitrosative challenge assays. A) The ability of wild type, ΔkpnGH and ΔkpnGHΩkpnGH to combat different levels of hydrogen peroxide was measured by disc diffusion assay. B) Survival of wild type, ΔkpnGH and ΔkpnGHΩkpnGH strains monitored upon exposure to H2O2 at 0.07894 mM, 0.7894 mM, 1.5788 mM, 2.3682 mM and 3.1576 mM. After 1 h of treatment with 0.07894 mM hydrogen peroxide, only 42% of ΔkpnGH cells survived in comparison to 91% of the wild-type cells. C) Growth pattern of wild type, ΔkpnGH and ΔkpnGHΩkpnGH in the presence of SNP. The growth kinetics of kpnGH exhibited 3.024-fold (±0.032; P = 0.000216), 3.38-fold (±0.072; P = 0.000241), 2.58-fold (±0.066; P = 0.000232), 1.702-fold (±0.012; P = 0.000141), 2.194-fold (±0.054; P = 0.000581), 1.132-fold (±0.076; P = 0.0002814) reduced growth compared to WT strain in LB at 0 mM, 5 mM, 10 mM, 15 mM, 20 mM and 25 mM respectively. The mean values of three independent experiments have been used for plotting the graph. D) Growth pattern of wild type, ΔkpnGH and ΔkpnGHΩkpnGH in the presence of NaNO2. The growth kinetics of kpnGH exhibited 2.88-fold (±0.045; P = 0.000115), 1.838-fold (±0.087; P = 0.000316), 1.629-fold (±0.026; P = 0.000211), 1.643-fold (±0.097; P = 0.000510), 1.454-fold (±0.032; P = 0.000409), 1.602-fold (±0.74; P = 0.000311) reduced growth compared to WT strain in LB at 0 mM, 5 mM, 10 mM, 15 mM, 20 mM and 25 mM respectively. The mean values of three independent experiments have been used for plotting the graph. (TIF) [file pone.0096288.s002.tif]

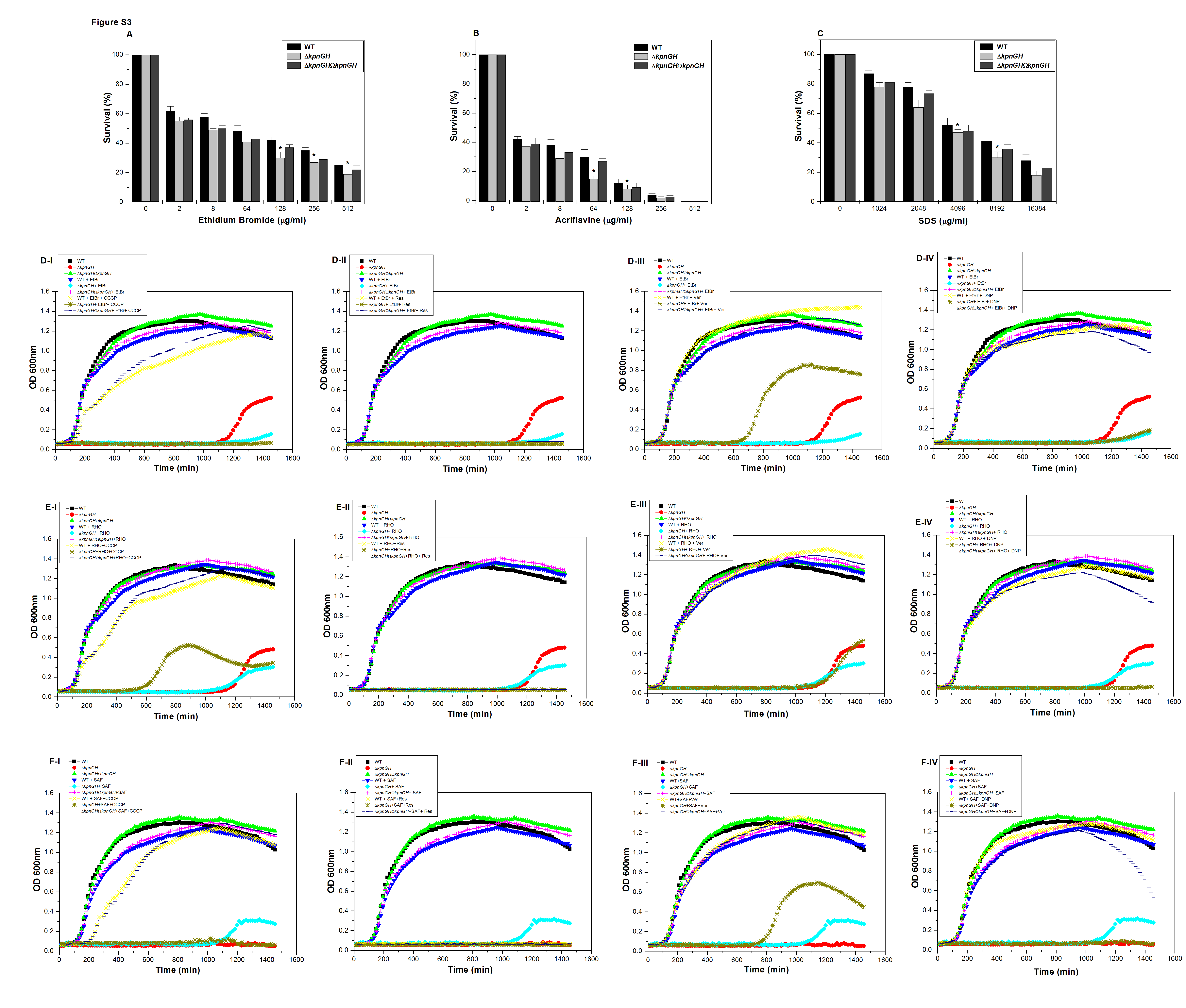

Supplement: Figure S3 — Susceptibility and Growth inactivation assays towards different dyes and detergent. A) Sensitivity of wild type, ΔkpnGH and ΔkpnGHΩkpnGH towards EtBr when cells were exposed to different concentrations of dye (2 µg/ml, 4 µg/ml, 64 µg/ml, 128 µg/ml, 256 µg/ml and 512 µg/ml). The data is the means of measurements made in triplicate and performed three times. B) Sensitivity of wild type, ΔkpnGH and ΔkpnGHΩkpnGH towards acriflavine when cells were exposed to different concentrations of dye (2 µg/ml, 4 µg/ml, 64 µg/ml, 128 µg/ml, 256 µg/ml and 512 µg/ml). The data is the means of measurements made in triplicate and performed three times. C) Sensitivity of wild type, ΔkpnGH and ΔkpnGHΩkpnGH towards SDS when cells were exposed to different concentrations of detergent (1024 µg/ml, 2048 µg/ml, 4096 µg/ml, 8192 µg/ml, 16834 µg/ml). The data is the means of measurements made in triplicate and performed three times. D) Growth inactivation assay using wild type, ΔkpnGH and ΔkpnGHΩkpnGH in the presence of EtBr. Growth pattern in absence of any EtBr or inhibitor is included as control. The growth rate of ΔkpnGH in the presence of 2.0 µg/ml EtBr, was >12 fold lesser when compared to that of WT (P = 0.000477). A decrease in growth was observed in kpnGH mutant (15.18-fold; P = 0.000697), (1.39-fold; P = 0.000775), (6.32-fold; P = 0.000681) and (15.06-fold; P = 0.000174) in the presence of CCCP (I), reserpine (II), verampamil (III) and 2, 4 DNP (IV) respectively. The mean values of three independent experiments have been used for plotting the graph. E) Growth inactivation assay using wild type, ΔkpnGH and ΔkpnGHΩkpnGH in the presence of rhodamine. Growth pattern in absence of any rhodamine or inhibitor is included as control. The growth rate of ΔkpnGH in the presence of 2.0 µg/ml rhodamine, was >17.5 fold lesser when compared to that of WT (P = 0.000153). A decrease in growth was observed in kpnGH mutant (10.476-fold; P = 0.000234), (1.39102-fold; P = 0.000796), (8.13-fold; P = 0.0001 [file pone.0096288.s003.tif]

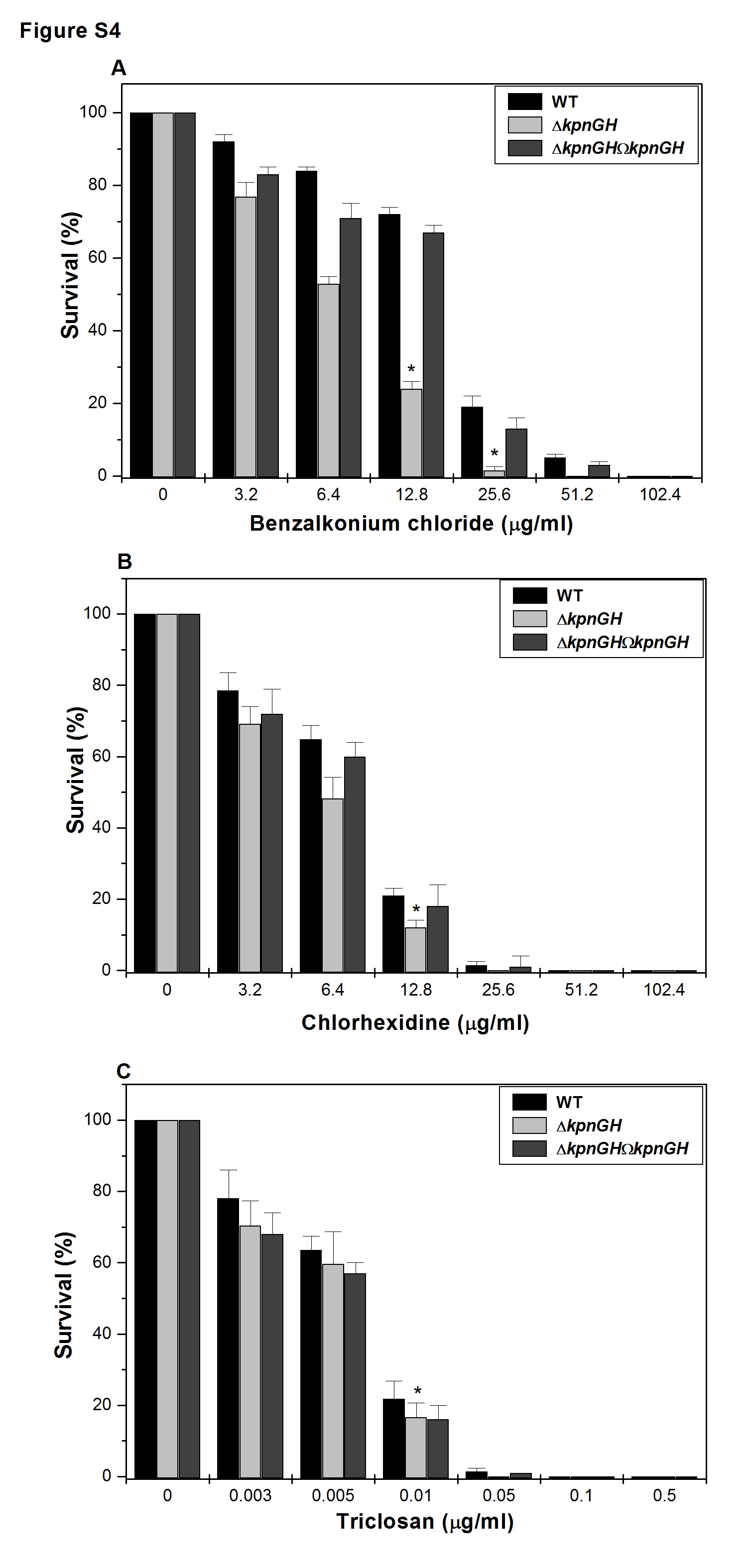

Supplement: Figure S4 — Disinfectant challenge assays. Survival of wild type, ΔkpnGH and ΔkpnGHΩkpnGH in the presence of different concentrations (µg/ml) of A) benzalkonium chloride (3.2, 6.4, 12.8, 25.6, 51.2, 102.4), B) chlorhexidine (3.2, 6.4, 12.8, 25.6, 51.2, 102.4), C) triclosan (0.003, 0.005, 0.01, 0.05, 0.1, 0.5). The percent survival was calculated by comparison to the numbers of viable cells obtained in LB medium alone. *, Significant difference (P<0.05, Student t test). (TIF) [file pone.0096288.s004.tif]

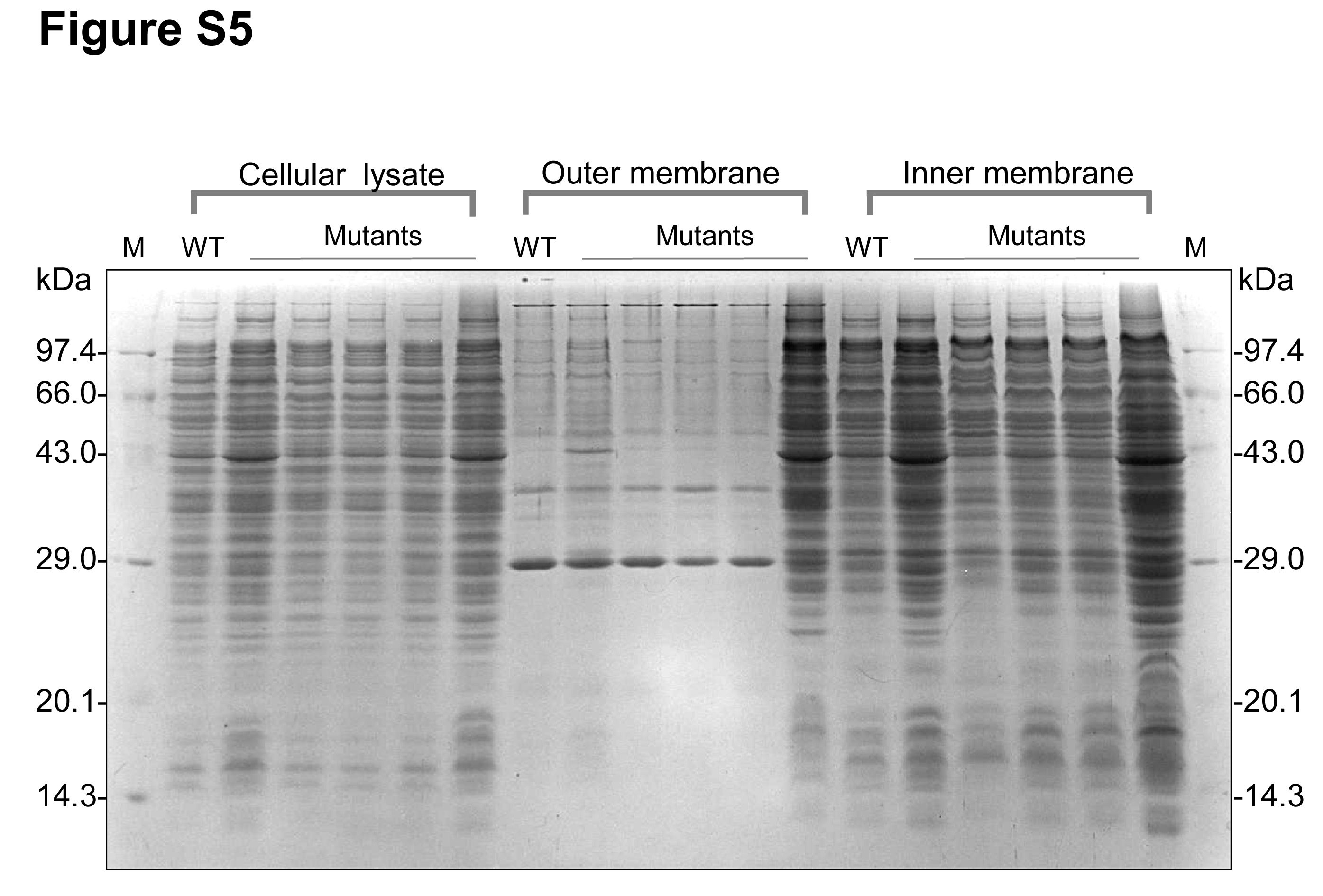

Supplement: Figure S5 — Protein profiling of WT and kpnGH mutants. Membrane protein profiles were compared between the WT strain, and kpnGH mutant. About 20 µg of total protein lysate was loaded in an order as follows; lane 2: WT, lane 3: kpnGH mutant 1, lane 4: kpnGH mutant 2, lane 5: kpnGH mutant3, lane 6: kpnGH mutant 4, lane 7: kpnGH mutant 5 respectively. A similar amount of outer membrane fractions was loaded in an order as follows; lane 8: WT, lane 9: kpnGH mutant 1, lane 10: kpnGH mutant 2, lane 11: kpnGH mutant3, lane 12: kpnGH mutant 4, lane 13: kpnGH mutant 5 respectively. The inner membrane fractions was loaded in an order as follows; lane 14: WT, lane 15: kpnGH mutant 1, lane 16: kpnGH mutant 2, lane 17: kpnGH mutant3, lane 18: kpnGH mutant 4, lane 19: kpnGH mutant 5 respectively. Equal protein concentrations were separated by SDS-PAGE with a 5% stacking gel and a 12% separating gel and stained with coomassie brilliant blue. Lane M has molecular weight standards. (TIF) [file pone.0096288.s005.tif]
